# Modeling Group B *Streptococcus* and Blood-Brain Barrier Interaction by Using Induced Pluripotent Stem Cell-Derived Brain Endothelial Cells

**DOI:** 10.1128/mSphere.00398-17

**Published:** 2017-11-01

**Authors:** Brandon J. Kim, Olivia B. Bee, Maura A. McDonagh, Matthew J. Stebbins, Sean P. Palecek, Kelly S. Doran, Eric V. Shusta

**Affiliations:** a Department of Chemical and Biological Engineering, University of Wisconsin, Madison, Wisconsin, USA; b Department of Immunology and Microbiology, University of Colorado School of Medicine, Aurora, Colorado, USA; University of Kentucky

**Keywords:** blood-brain barrier, group B *Streptococcus*, stem cells

## Abstract

Bacterial meningitis is a serious infection of the central nervous system (CNS) that occurs after bacteria interact with and penetrate the blood-brain barrier (BBB). The BBB is comprised of highly specialized brain microvascular endothelial cells (BMECs) that function to separate the circulation from the CNS and act as a formidable barrier for toxins and pathogens. Certain bacteria, such as *Streptococcus agalactiae* (group B *Streptococcus* [GBS]), possess the ability to interact with and penetrate the BBB to cause meningitis. Modeling bacterial interaction with the BBB *in vitro* has been limited to primary and immortalized BMEC culture. While useful, these cells often do not retain BBB-like properties, and human primary cells have limited availability. Recently, a human induced pluripotent stem cell (iPSC)-derived BMEC model has been established that is readily renewable and retains key BBB phenotypes. Here, we sought to evaluate whether the iPSC-derived BMECs were appropriate for modeling bacterial interaction with the BBB. Using GBS as a model meningeal pathogen, we demonstrate that wild-type GBS adhered to, invaded, and activated the iPSC-derived BMECs, while GBS mutants known to have diminished BBB interaction were attenuated in the iPSC-derived model. Furthermore, bacterial infection resulted in the disruption of tight junction components ZO-1, occludin, and claudin-5. Thus, we show for the first time that the iPSC-derived BBB model can be utilized to study BBB interaction with a bacterial CNS pathogen.

**IMPORTANCE** Here for the first time, human iPSC-derived BMECs were used to model bacterial interaction with the BBB. Unlike models previously used to study these interactions, iPSC-derived BMECs possess robust BBB properties, such as the expression of complex tight junctions that are key components for the investigation of bacterial effects on the BBB. Here, we demonstrated that GBS interacts with the iPSC-derived BMECs and specifically disrupts these tight junctions. Thus, using this BBB model may allow researchers to uncover novel mechanisms of BBB disruption during meningitis that are inaccessible to immortalized or primary cell models that lack substantial tight junctions.

## Abstract (precis)

Here for the first time, human iPSC-derived BMECs were used to model bacterial interaction with the BBB. Unlike models previously used to study these interactions, iPSC-derived BMECs possess robust BBB properties, such as the expression of complex tight junctions that are key components for the investigation of bacterial effects on the BBB. Here, we demonstrated that GBS interacts with the iPSC-derived BMECs and specifically disrupts these tight junctions. Thus, using this BBB model may allow researchers to uncover novel mechanisms of BBB disruption during meningitis that are inaccessible to immortalized or primary cell models that lack substantial tight junctions.

## INTRODUCTION

Bacterial meningitis is a serious, life-threatening infection of the central nervous system (CNS) and a major cause of death and disability worldwide, with a disproportionate number of cases involving children (1–3). While current antibiotic therapy has transformed bacterial meningitis from a uniformly fatal condition into an often-curable one, mortality remains between 5 and 10%, with permanent neurologic sequelae occurring in 5 to 40% of survivors, depending on the patient’s age and the pathogen (1–3). To cause meningitis, bacteria must gain access to the bloodstream and replicate to a high level, causing bacteremia (1). Following bacteremia, bacteria must then interact with and penetrate the blood-brain barrier (BBB) to gain access to the central nervous system (CNS). The specialized brain microvascular endothelial cells (BMECs) that comprise the BBB respond to these bacterial interactions with a cellular immune response and contribute to the disease progression (1–3).

*Streptococcus agalactiae* (group B *Streptococcus* [GBS]) is a Gram-positive, non-spore-forming bacterium that is the leading cause of neonatal meningitis and is an emerging pathogen in specific adult populations (1, 4, 5). Although advancements have been made in diagnosis and therapy, death still occurs in up to 10% of cases, with 25 to 50% of surviving infants exhibiting permanent neurological sequelae (1, 4, 5). More recently, much work has been conducted to determine bacterial virulence factors that contribute to interaction with the BBB and allow bacterial access to the CNS. Bacterial surface-expressed factors, such as lipoteichoic acid (LTA) (6), pilus components (PilA, PilB, and PilC) (7, 8), serine-rich repeat proteins (Srr) (9–11), streptococcal fibronectin binding factor (SfbA) (12), fibrinogen-binding protein (FbsA) (13), and hypervirulent GBS adhesin (HvgA) (14), have all been demonstrated to promote direct association with the BBB. Additionally, regulatory two-component signal transduction systems, such as CovR/S (15), and CiaR/H (16), have been implicated in the ability to regulate virulence factors that contribute to the pathogenesis of GBS meningitis. Studies have also been conducted to determine the BBB response to GBS. During infection, immortalized BMECs have been shown to downregulate tight junctions through the induction of host transcription factor Snail1, a known repressor of tight junction components. This response resulted in a significant loss of barrier function during infection, which was dependent on Snail1 expression (17). Previous studies have also demonstrated that GBS infection of immortalized BMECs upregulates proinflammatory chemokines and cytokines that act to orchestrate the recruitment and activation of neutrophils and enhance their survival (1, 7, 18). The recruitment of neutrophils has been linked to further BBB destruction during infection, and bacterial determinants that include CovR/S, PilA, and β-hemolysin/cytolysin contribute to this process (7, 15, 18). Together, these factors promote GBS penetration of the BBB, allowing access to the CNS and the development of meningitis.

The BBB is comprised of highly specialized BMECs that serve to separate the brain from the circulation and, along with cells of the neurovascular unit (NVU), maintain CNS homeostasis (19–21). BMECs express a spectrum of nutrient transporters and multidrug efflux transporters while also displaying intercellular tight junctions and low endocytosis rates (19–21). To study the BBB, researchers have relied on complementary *in vivo* and *in vitro* techniques. Animal models have been utilized to examine bacterial interactions with the BBB in the context of the full CNS microenvironment (6, 22–28); however, these are inherently nonhuman models and subject to interspecies differences (29). Primary cell culture of animal and human BMECs has been employed (30–33); however, after removing BMECs from the brain microenvironment, they routinely lose BBB characteristics (29, 34, 35). Immortalized human BMECs offer a facile human-based model (29, 34–36), yet many of these cell lines lack critical BBB properties, such as high transendothelial electrical resistance (TEER) and complex tight junctions (29, 34–36). Recently, induced pluripotent stem cells (iPSCs) have offered the prospect of renewable BBB models with superior barrier properties (29, 34, 37–40). The model has been used to examine drug delivery, genetic human disease, and ischemic stroke, but it has not yet been evaluated for its applicability to infectious disease (41–44). Here, we demonstrate that an iPSC-derived BMEC model can be used to examine host-pathogen interactions using the meningeal pathogen GBS. These results motivate the application of this model in infectious disease research.

## RESULTS

###  Wild-type GBS interacts with iPSC-derived BMECs.

**FIG 1:**
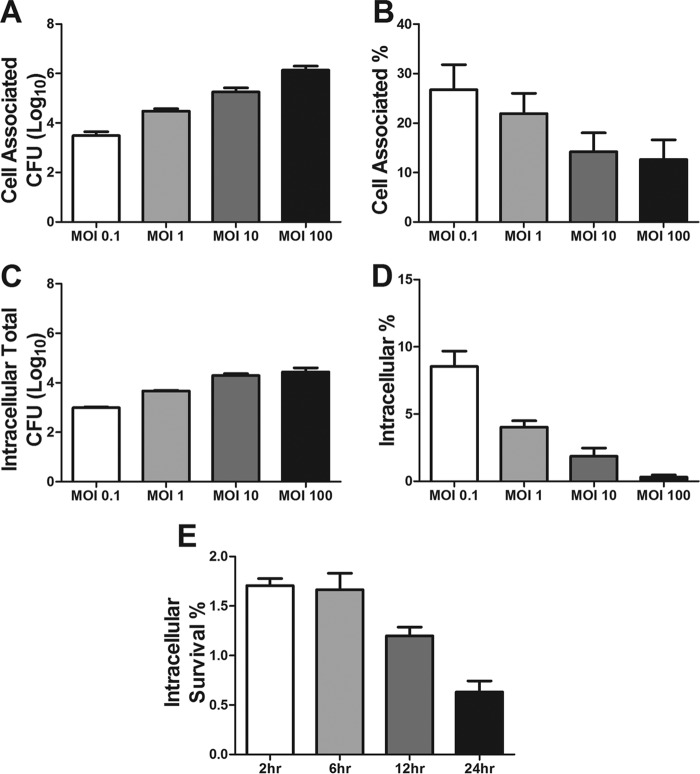
Interaction of group B *Streptococcus* with iPSC-derived BMECs. (A and B) Adherence of wild-type GBS to iPSC-derived BMECs over a range of MOIs, expressed as total CFU recovered (A) and percentage of initial inoculum (B). (C and D) Invasion of wild-type GBS into iPSC-derived BMECs, presented as total recovered CFU (C) and percentage of initial inoculum (D). (E) Survival of intracellular GBS, sampled from 2 to 24 h after infection of iPSC-derived BMECs at an MOI of 10, presented as the percentages of the initial inoculum. Data are presented as mean values from three independent iPSC-derived BMEC differentiations conducted in triplicate. Error bars represent SEM.

Previous studies have shown that GBS has the ability to interact with and invade immortalized human brain endothelial cells (hBMECs) (1, 3, 4, 14, 31). Thus, we sought to determine if iPSC-derived BMECs could be used to model these interactions. The iPSC-derived BMECs were differentiated as previously described and were shown to express the expected markers and respond to astrocyte cues (Fig. S1A to G) (29, 40). In addition, the iPSC-derived BMECs express β1 integrin, a receptor for GBS virulence factors (Fig. S1H) (7, 12). Wild-type serotype III, hypervirulent, multilocus sequence type 17 (MLST-17) GBS strain COH1 was used to examine GBS interactions with iPSC-derived BMECs. Confluent iPSC-derived BMEC monolayers were infected with GBS to assess bacterial attachment and intracellular invasion. The numbers of total cell-associated and intracellular bacteria recovered increased proportionally with the multiplicity of infection (MOI) of the original inoculum (Fig. 1A and C). However, as the MOI increased, the percentage of adherent or intracellular GBS recovered relative to the original inoculum decreased in a stepwise fashion (Fig. 1B and D), indicating that bacterial attachment and uptake mechanisms are saturable. These results were specific to GBS, as the cell-associated and intracellular populations of the nonpathogenic bacterium *Lactococcus lactis* were significantly less than those observed for GBS (Fig. S2). To determine if GBS is able to survive intracellular uptake into iPSC-derived BMECs, we performed a modified invasion assay in which the numbers of intracellular GBS bacteria were quantified at different time points after the addition of antibiotics to eliminate extracellular bacteria. As shown by the results in Fig. 1E, GBS persisted in iPSC-derived BMECs for up to 6 h and then exhibited a decrease in intracellular survival over time. Overall, these data demonstrate that GBS can specifically interact with iPSC-derived BMECs and are consistent with results obtained in immortalized hBMECs and other cell types (16, 31, 45, 46).

10.1128/mSphere.00398-17.1 FIG S1  Characterization of DF19-9-11-derived BMECs. Representative immunofluorescence images of iPSC-derived BMECs after differentiation. (A to F) CD31 (pecam-1) (A), glut-1 (B), claudin-5 (C), occludin (D), BCRP (E), and PGP (F). Scale bar represents 50 μm. (G) Maximum TEER of iPSC-derived BMECs in monoculture versus iPSC-derived BMECs in coculture with iPSC-derived astrocytes. (H) Representative flow cytometry histograms of β1 integrin expression on iPSC-derived BMEC surface. Data are presented as mean values of three independent differentiations conducted in triplicate. Error bars represent SEM. Student’s *t* test was used to determine significance. **, *P* < 0.01. Download FIG S1, PDF file, 0.5 MB. Copyright © 2017 Kim et al. 2017 Kim et al. This content is distributed under the terms of the Creative Commons Attribution 4.0 International license.

10.1128/mSphere.00398-17.2 FIG S2  *L. lactis* interaction with iPSC-derived BMECs. Adherence (A) and invasion (B) assays of wild-type GBS versus wild-type *L. lactis* at an MOI of 10. Data are presented as mean values of the combined data from two independent differentiations conducted in triplicate. Error bars represent SD. Student’s *t* test was used to determine significance. **, *P* < 0.01; ***, *P* < 0.001. Download FIG S2, PDF file, 0.02 MB. Copyright © 2017 Kim et al. 2017 Kim et al. This content is distributed under the terms of the Creative Commons Attribution 4.0 International license.

### GBS virulence factors contribute to interaction with iPSC-derived BMECs.

**FIG 2:**
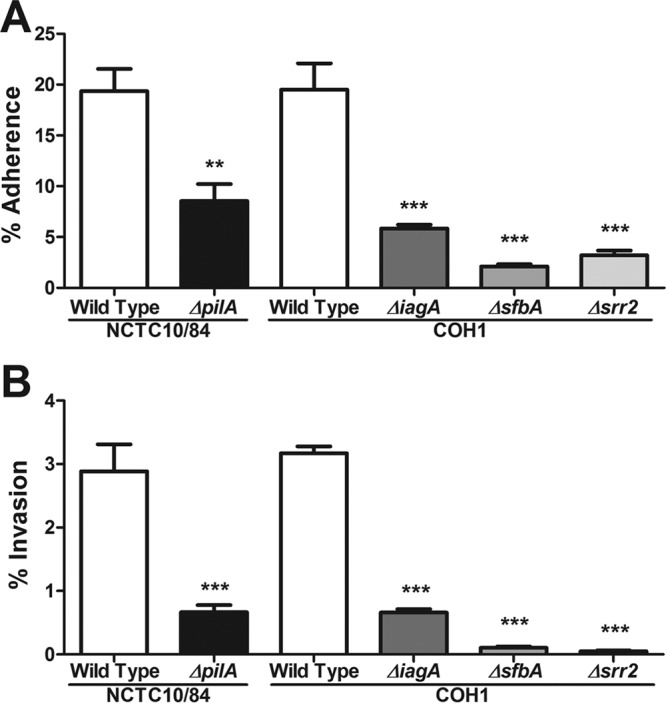
Contribution of GBS virulence factors to interaction with iPSC-derived BMECs. Adherence (A) and invasion (B) of wild-type GBS strains compared to those of GBS mutants lacking the virulence factors *pilA*, *iagA*, *sfbA*, and *srr2*. Data are presented as mean values from three independent iPSC-derived BMEC differentiations conducted in triplicate. Error bars represent SEM. Student’s *t* test was used to determine the significance of the difference between WT NCTC10/84 and the *ΔpilA* mutant. ANOVA was used to determine the significance of the difference between WT COH1 and the *ΔiagA*, *ΔsfbA*, and *Δsrr2* mutants. **, *P* < 0.01; ***, *P* < 0.001.

Recently, much work has been conducted to identify and characterize virulence factors that promote GBS interaction with the BBB (6–8, 11–14, 47). To determine whether several well characterized GBS virulence factors affected interactions with iPSC-derived BMECs, a cohort of mutants were examined for their ability to promote attachment and invasion of the iPSC-derived BMECs. We selected GBS factors that have been shown to be critical for BBB interaction and the pathogenesis of GBS meningitis. Specifically, we chose surface-expressed factors, including PilA (7, 8), SfbA (47), and Srr2 (9, 10), all of which contribute to GBS interaction with extracellular matrix (ECM) components acting to bridge the bacteria to host ECM receptors (1). Additionally, we analyzed the contribution of an invasion-associated gene (*iagA*) whose product acts to anchor lipoteichoic acid (LTA) to the cell surface and promote bacterial uptake into the brain endothelium (1, 6–12). We observed that infection of iPSC-derived BMECs with these previously generated and described GBS mutant strains resulted in significant decreases in the numbers of adherent and/or intracellular bacteria recovered compared to the results for the wild-type parental GBS strains recovered (Fig. 2). Thus, these data demonstrate that these mutant GBS strains exhibited the expected attenuated attachment and invasion phenotypes using iPSC-derived BMECs.

### iPSC-derived BMECs are activated in response to GBS infection.

**FIG 3:**
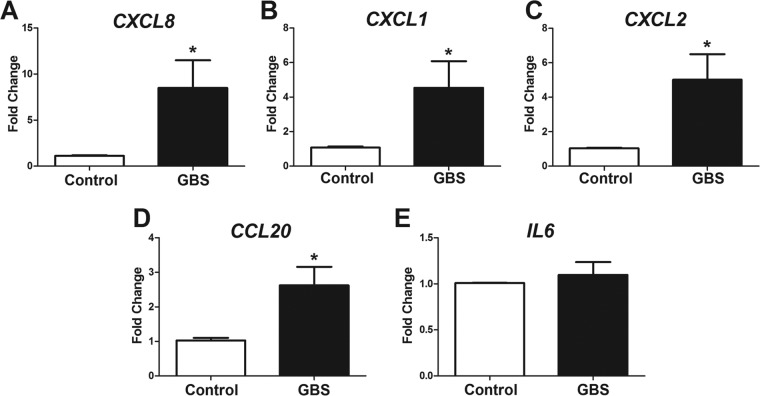
GBS induced activation of iPSC-derived BMECs. Quantitative PCR was performed on iPSC-derived BMECs with or without wild-type GBS infection at an MOI of 10 for 5 h. Neutrophil chemoattractant-coding genes *CXCL8* (IL-8) (A), *CXCL1* (CXCL-1) (B), and *CXCL2* (CXCL-2) (C) and proinflammatory cytokine-coding genes *CCL20* (CCL-20) (D) and *IL6* (IL-6) (E) were evaluated. Data are presented as mean fold changes compared to the results for uninfected controls for at least three independent iPSC-derived BMEC differentiations conducted in triplicate. Error bars represent SEM. Student’s *t* test was used to determine significance. *, *P* < 0.01.

Previous reports have shown that the major proinflammatory BBB response to GBS infection is the upregulation of chemokines and cytokines that promote neutrophilic influx (7, 18). Thus, the transcript expression of key chemokines in iPSC-derived BMECs was evaluated following GBS infection. Wild-type GBS at an MOI of 10 was used to infect iPSC-derived BMECs for 5 h, and no loss in cell viability was observed under these conditions (Fig. S3). We observed that *CXCL8* (encoding interleukin-8 [IL-8]), *CXCL1*, and *CXCL2*, as well as *CCL20*, encoding neutrophil chemoattractants, were significantly upregulated in response to GBS infection compared to their levels in the noninfected control (Fig. 3A to D). In contrast, the expression of the gene encoding the global proinflammatory cytokine IL-6 was unchanged in response to GBS infection (Fig. 3E). Together, these data show that key proinflammatory chemokines are induced in iPSC-derived BMECs during GBS infection.

10.1128/mSphere.00398-17.3 FIG S3  Viability of iPSC-derived BMEC during GBS infection. Trypan blue exclusion counts of iPSC-derived BMEC uninfected or infected with wild-type GBS for 5 h. Data are presented as values for triplicate wells. Error bars represent SD. Download FIG S3, PDF file, 0.01 MB. Copyright © 2017 Kim et al. 2017 Kim et al. This content is distributed under the terms of the Creative Commons Attribution 4.0 International license.

### GBS infection disrupts tight junctions of iPSC-derived BMECs.

**FIG 4:**
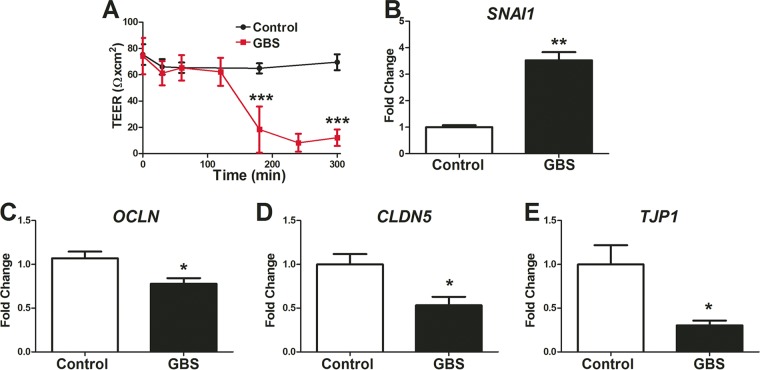
GBS induced BBB disruption on iPSC-derived BMECs. (A) TEER profile during wild-type GBS infection at an MOI of 10. (B to E) Quantitative PCR evaluation of iPSC-derived BMECs infected with wild-type GBS at an MOI of 10 for 5 h. Transcripts of Snail1 (*SNAI1*) (B), occludin (*OCLN*) (C), claudin-5 (*CLDN5*) (D), and ZO-1 (*TJP1*) (E) were monitored. qPCR data are presented as mean fold changes compared to the results for uninfected controls for at least three independent iPSC-derived BMEC differentiations conducted in triplicate. Error bars represent SEM. Student’s *t* test was used to determine significance. *, *P* < 0.05; **, *P* < 0.01; ***, *P* < 0.001.

**FIG 5:**
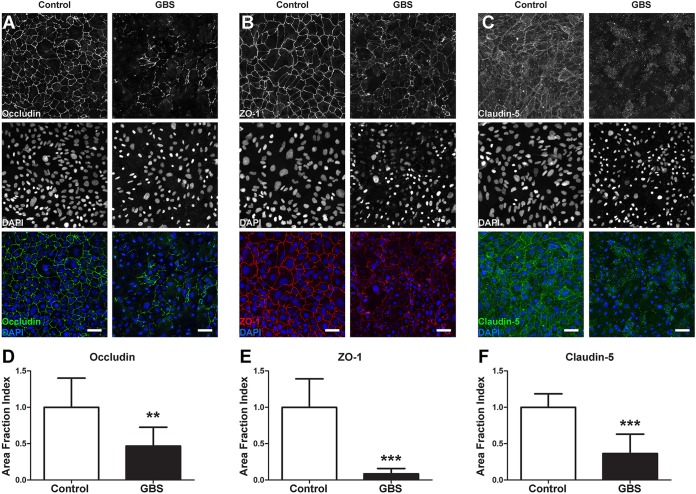
GBS induced tight junction disruption in iPSC-derived BMECs. iPSC-derived BMECs were stained for tight junction proteins following GBS infection, and the results were compared to those for the uninfected control. (A to C) Representative images of occludin (A), ZO-1 (B), and claudin-5 (C) staining. Scale bars represent 50 μm. (D to F) Area fraction indices of occludin (D), ZO-1 (E), and claudin-5 (F) staining after GBS infection. Area fraction index data are presented as the mean values of at least six independent images taken from at least two independent differentiations, with three images taken per differentiation. Error bars represent SD. Student’s *t* test was used to determine significance. *, *P* < 0.05; **, *P* < 0.01; ***, *P* < 0.001.

It is known that GBS infection can affect tight junction components and BBB barrier function (17). To determine whether iPSC-derived BMECs were affected by GBS interactions, we used TEER to measure barrier integrity. After 3 h post-GBS infection, we observed a dramatic decrease in the barrier function of iPSC-derived BMECs (Fig. 4A). Tight junction dysfunction during GBS infection was recently reported to be linked to the expression of the tight junction transcriptional repressor Snail1 (*SNAI1*) (17). Consistent with this finding, upregulation of *SNAI1* transcript expression was observed in iPSC-derived BMECs following GBS infection (Fig. 4B). Transcripts for genes encoding tight junction proteins occludin (*OCLN*), claudin-5 (*CLDN5*), and ZO-1 (*TJP1*) were all also decreased during GBS exposure (Fig. 4C to E). The transcript downregulation corresponded to a loss of tight junction complexes, as immunostaining revealed that occludin, claudin-5, and ZO-1 proteins were noticeably discontinuous or absent from cell-cell junctions following infection (Fig. 5A to C). Quantitation using the area fraction index indicated that this decrease was significant compared to the results for the uninfected control (Fig. 5D to F). In contrast, an immortalized hBMEC cell line exhibited a lack of tight junction continuity and did not express claudin-5 (Fig. S4A to E), which is consistent with previous studies using this cell line to examine GBS infection (17). Taken together, these results demonstrate that GBS infection results in the disruption of tight junctions in iPSC-derived BMECs.

10.1128/mSphere.00398-17.4 FIG S4  Tight junctions on immortalized hBMECs. Immortalized hBMECs were immunolabeled for tight junction proteins following GBS infection, and the results were compared to those for the uninfected control. (A to C) Representative images of occludin (A), ZO-1 (B), and claudin-5 (C) staining. Scale bar represents 50 µm. (D and E) Western blot analysis of claudin-5 abundance following GBS infection compared to the results for an uninfected control in immortalized hBMECs (D) and iPSC-derived BMECs (E). Images are representative of at least two experiments on biological replicates (A to D) or of three independent differentiations (E), conducted in triplicate. Download FIG S4, PDF file, 2.6 MB. Copyright © 2017 Kim et al. 2017 Kim et al. This content is distributed under the terms of the Creative Commons Attribution 4.0 International license.

## DISCUSSION

Here, we demonstrate for the first time the use of iPSC-derived BMECs to model the critical steps of bacterial-brain endothelial interactions that lead to BBB penetration and the development of bacterial meningitis. Until now, *in vitro* studies of bacterium-BBB interactions have largely relied on immortalized human BMECs (hBMECs) (6–8, 12, 15, 17, 18, 24, 30, 31, 45, 47–52). While the immortalized hBMECs offered the ability to begin understanding the molecular interactions leading to BBB penetration, the model itself lacks important BBB properties, such as the proper expression and localization of tight junction proteins (Fig. S4) (17). More recently, other immortalized human BBB models, such as the human cerebral microvascular endothelial cell (hCMEC) line hCMEC/D3, have been developed. In contrast to the immortalized hBMECs described here, hCMEC/D3s do express junctional claudin-5; however, occludin expression is discontinuous, leading to modest TEERs (~40 Ω × cm^2^) that are not substantially improved upon coculture with astrocytes (35, 36, 53–56). Similar to immortalized human BMEC lines, the iPSC-derived BMEC model offers a reliable and scalable method of generating BMECs that express BBB markers. iPSC-derived BMECs offer the additional advantages of continuous tight junctions, barrier formation, and elevated TEER in response to astrocyte cues (Fig. S1) (29, 38, 40, 57). Furthermore, the addition of retinoic acid to the iPSC-derived BMEC differentiation can greatly elevate TEER values, to physiological levels (38, 40). Our results suggest that the iPSC-derived BMEC model can be utilized to study bacterial attachment, invasion, immune activation, and tight junction disruption using GBS as a model meningeal pathogen. In addition, our data compare well with published data regarding attachment and invasion percentages using the immortalized BMEC models. Previous work has demonstrated a range of GBS attachment of between 15 and 28%, with intracellular CFU in the range of 2.5 to 4% (11, 12, 47); in the present study, we observed comparable percentages. This new model may also be helpful for future studies examining other bacterial meningeal pathogens, such as *Streptococcus pneumoniae* (pneumococcus), *Neisseria meningitidis* (meningococcus), and *Escherichia coli* strain K1.

The physical interaction between GBS and brain endothelial cells has been characterized previously using immortalized human BMECs (1). GBS possesses the ability to adhere to and invade brain endothelial cells through the use of a variety of virulence factors, such as the PilA, SfbA, Srr2, and IagA proteins (6–12). Our results demonstrate that these factors also contribute to bacterial interaction with the iPSC-derived BMECs. Many of the GBS virulence factors we examined are proteins that promote bacterial interaction with ECM components, including collagen (for PilA), fibronectin (for SfbA), and fibrinogen (for Srr2). These interactions are thought to allow GBS to bridge to host cell receptors directly, promoting bacterial attachment and invasion of multiple host cell types, including brain endothelial cells (7, 9, 12). Here, we show that these adhesins also promote GBS interaction with iPSC-derived BMECs, suggesting that the iPSC-derived BMECs maintain similar cellular receptors necessary for GBS attachment. As it has been previously thought that attachment would precede invasion, it is then unsurprising that adhesion deficiencies, such as in the case of PilA, may also result in a decrease in invasion. Studies have also described a role for IagA, a glycosyltransferase that acts to generate the glycolipid anchor for LTA, in the pathogenesis of GBS using immortalized BMECs *in vitro* and a mouse model of GBS infection. Our results support these previous findings, as the Δ*iagA* mutant was less invasive with the iPSC-derived BMECs, although we observed that the Δ*iagA* mutant also had reduced adherent properties. It is unknown at this point whether the deletion of *iagA* only impacts anchored LTA or whether other surface factors are disrupted in the Δ*iagA* mutant that may impact GBS-BBB interactions. The work characterizing GBS SfbA in immortalized hBMECs has demonstrated its contribution to bacterial entry (12), while we observed a role for both attachment and invasion of iPSC-derived BMECs. Interestingly, SfbA is a homolog of the pneumococcal adhesin PavA, previously shown to contribute to pneumococcal adherence to BMECs (58, 59), and therefore, it is possible that SfbA could be contributing to GBS adherence, which is better detected in the iPSC-derived model. Additionally SfbA has been reported to contribute to GBS adherence to fibronectin (12), and the binding of extracellular matrix as a means to adhere to host BBB has been discussed previously (7, 13, 49). Regardless, our results demonstrate that known GBS mutants are attenuated in their interaction with iPSC-derived BMECs, suggesting that in general, similar mechanisms for bacterial-host cell interactions are preserved in the iPSC-derived-BMEC model.

Bacterial activation of the brain endothelium has been hypothesized to contribute to disease progression through the upregulation of cytokines and chemokines that attract circulating leukocytes, specifically neutrophils (1–5). Previous work has demonstrated that the recruitment of neutrophils contributes to further BBB destruction in murine models of GBS meningitis (7). As expected, in response to GBS infection, iPSC-derived BMECs upregulate *CXCL8* (IL-8), *CXCL1*, and *CXCL2*, encoding potent neutrophil chemoattractants. Our findings agree with published results on the immortalized BMEC models, where the expression levels of *CXCL8*, *CXCL1*, and *CXCL2* were upregulated between 6- and 15-fold (7). Interestingly, however, we did not observe upregulation of *IL6*, encoding the global proinflammatory cytokine IL-6. It is possible that IL-6 may be generated from other sources besides the brain endothelium *in vivo*, given that it is detected at high levels in the sera of infected animals (7). Further investigation is needed to determine the overall transcriptional profile of iPSC-derived BMECs during GBS infection. However, GBS infection of iPSC-derived BMECs induced a set of chemokines that act to orchestrate neutrophil recruitment and activation.

We recently demonstrated that GBS is able to induce the expression of the transcriptional repressor of tight junctions, Snail1, which contributes to tight junction disruption in immortalized hBMECs during GBS infection (17). In agreement with these findings, GBS infection of iPSC-derived BMECs upregulated Snail1 and substantially decreased the tight junctional continuity and resultant barrier properties. Furthermore, our previous work examining tight junction disruption was limited by the use of immortalized human BMEC cell lines that did not express claudin-5 and lacked continuous occludin staining and, thus, lacked an optimal barrier phenotype (Fig. S4) (17). The iPSC-derived BMECs express claudin-5 and have properly localized occludin and, thus, allow for the much more relevant analysis of tight junction function during GBS infection.

*In vivo*, BMECs are supported by a number of other CNS cell types, such as the astrocytes, neurons, and pericytes that make up the NVU. Currently, little is known about the contribution of astrocytes, neurons, and pericytes to BBB function during meningitis. Models combining iPSC-derived neurons and astrocytes along with iPSC-derived BMECs have been reported by us and others (29, 34, 35, 37, 38, 57, 60). Combined with this report establishing iPSC-derived BMECs as a model to study bacterial meningitis, these tools will likely support future studies examining the role of other NVU cell types and their contributions during infection.

## MATERIALS AND METHODS

### Bacterial strains and cell lines used.

Group B *Streptococcus* (GBS; *Streptococcus agalactiae*) hypervirulent clinical isolates COH1 (serotype III, multilocus sequence type 17 [MLST-17]) (61) and NCTC10/84 (serotype V, MLST-26) (62) were used. COH1 *ΔiagA* (6), *Δsrr2* (63, 64), and *ΔsfbA* (12) and NCTC10/84 *ΔpilA* (8) mutants have been described previously. GBS strains were all grown in Todd-Hewitt broth (THB) at 37°C. *Lactococcus lactis* was grown in M17 medium at 30°C (8). iPSC line DF19-9-11 (WiCell) was chosen for this study as this iPSC line was not generated through the use of viral integration vectors, eliminating the potential for inherent antiviral or anti-inflammatory responses. DF19-9-11 cells were grown in mTeSR1 medium (WiCell) that was changed daily and maintained on Matrigel (WiCell)-coated plates (Corning) consistent with previously published methods (29, 38–40, 57). Immortalized hBMECs (a gift of Kwang Sik Kim and Monique Stins, Johns Hopkins University, Baltimore, MD) were cultured in RPMI 1640 containing 10% fetal bovine serum (FBS), 10% NuSerum, and 1% nonessential amino acids as described previously (6, 7, 12, 17, 31).

### Generation of BMECs and astrocytes from iPSCs.

DF19-9-11 iPSCs were differentiated into BMECs according to methods published previously (29, 38–40, 57). Briefly, a single-cell suspension of iPSCs was seeded at a density of 10,000/cm^2^ onto Matrigel (WiCell) cell culture plates or flasks (Corning) and grown for 3 days. Differentiation was initiated by changing to UM medium (Dulbecco modified Eagle medium [DMEM]–F-12 medium plus 20% knockout serum replacement [KOSR], 1% minimal essential medium [MEM], 0.5% Glutamax, and 0.07% beta-mercaptoethanol) for 6 days, refreshing the medium daily. The medium was then changed to EC medium (human endothelial cell serum-free medium plus 1% platelet-poor plasma-derived serum [Fisher] and 500 ng/ml basic fibroblast growth factor [bFGF]) for 2 days. Finally, BMECs were purified on fibronectin- and collagen-coated plates or Transwell inserts (Corning, product number 3460). BMECs were analyzed for TEER using an EVOM II instrument (World Precision) and for the expression of endothelial markers as described previously (29, 40). The addition of retinoic acid during BMEC differentiation has been shown to elevate BBB properties, including TEER and vascular endothelial (VE)-cadherin expression (38, 39). However, since retinoic acid has been shown to have anti-inflammatory properties and could interfere with cellular responses to bacteria (65–69), it was not used. Astrocytes were generated as described previously (57).

### Infection assays.

iPSC-derived BMECs purified onto collagen-fibronectin-coated 24-well plates (Corning) were grown to a confluent monolayer. A multiplicity of infection (MOI) of 10 was used for all experiments unless noted specifically. Overnight cultures of GBS or *L. lactis* were grown in THB or M17, respectively, subcultured the following day, and grown to an optical density at 600 nm (OD_600_) of 0.4. Bacteria were spun down and washed in phosphate-buffered saline (PBS) prior to infecting BMECs. Enumeration of total cell-associated and intracellular CFU was performed as described previously (6, 7, 31). Briefly, for adherence, bacteria were incubated with BMECs for 30 min at 37°C and 5% CO_2_, followed by 5 washes in PBS. Mammalian cells were lysed in 0.025% Triton X-100 and plated in a dilution series onto THB plates (M17 for *L. lactis*) to determine bacterial loads. For invasion, bacteria were incubated at 37°C and 5% CO_2_ for 2 h, followed by 3 washes in PBS and incubation with antibiotic medium for an additional 2 h at 37°C and 5% CO_2_. After the second 2-h incubation, BMECs were lysed in 0.025% Triton X-100 and plated in a dilution series onto THB plates (M17 for *L. lactis*) to determine bacterial loads. Intracellular survival assays were run exactly like the intracellular assessment assays; however, cultures were incubated in antibiotic medium for extended periods of time as described previously (45). The following day, bacterial loads were determined by counting the colonies of countable dilutions and back calculating. Data are presented as total CFU recovered or as the percentage of the initial inoculum.

### RNA isolation and quantitative PCR.

iPSC-derived BMECs were infected with GBS for 5 h at 37°C and 5% CO_2_. Following infection, cell lysates were collected for RNA isolation in lysis buffer (PureLink RNA minikit; Life Technologies, Inc.). RNA was purified using the PureLink RNA minikit (Life Technologies, Inc.), and cDNA was generated utilizing the Vilo first-strand kit (Life Technologies, Inc.). SYBR green (Thermo Fisher) quantitative PCR (qPCR) for *CXCL8* (IL-8), *CXCL1*, *CXCL2*, *CCL20*, *IL6*, *GAPDH* (glyceraldehyde-3-phosphate dehydrogenase [GAPDH]), and *SNAI1* (Snail1) was conducted using previously described primers (6, 17, 24). TaqMan probes (Thermo Fisher) were used for detection of *TJP1* (ZO-1) (Hs0155186_m1), *CLDN5* (claudin-5) (Hs00533949_s1), *OCLN* (occludin) (Hs00170162_m1), and *GAPDH* (Hs02786624_g1). qPCR data were collected on a BioRad CFX96 thermocycler, and data are presented as fold change over the results for *GAPDH* using the cycle threshold (ΔΔ*C*_*T*_) calculation.

### Immunofluorescence and area fraction index calculations.

Immortalized hBMECs and iPSC-derived BMECs were either infected with GBS for 5 h at 37°C and 5% CO_2_ or left uninfected as controls. After infection, cells were fixed and stained exactly as reported previously (40), with the single exception that anti-ZO-1 antibody (catalog number 339100; Thermo Scientific) was used in place of the previously reported anti-ZO-1 antibody. Briefly, BMECs were fixed in 100% ice-cold methanol and stained overnight at 4°C. Secondary antibodies were utilized at a 1:200 dilution, and samples were visualized with an Olympus IX70 inverted fluorescence microscope using Nikon NIS image acquisition software. Fiji ImageJ was used to create merged images. Determination of the area fraction index of tight junction staining and continuity was performed as described previously (57). Using Fiji ImageJ, images were corrected for inconsistent fluorescence illumination using the Background Correction plugin. Next, the gray scale intensity profile was examined to determine a threshold value that minimizes background while maintaining the staining profile. Images were then converted to a binary image and processed using the outline filter to determine the perimeter of tight junction staining in pixels. Total pixels were normalized to the square root of the number of cells in the image.

### Flow cytometry.

To assay the expression of the β1 integrin, iPSC-derived BMECs were differentiated, fixed in 1% paraformaldehyde, and stained using a 1:1,000 dilution of anti-β1 integrin antibody (catalog number NB100-63255; Abcam, Inc.) in 0.5% bovine serum albumin (BSA) in PBS overnight at 4°C. The following day, fixed cells were washed in 0.5% BSA in PBS twice and stained with anti-mouse IgG Alexa Fluor 488 antibody (catalog number A-11001; Life Technologies, Inc.) for 1 h at room temperature. Cells were then washed twice in 0.5% BSA in PBS and run on a BD Accuri C6 flow cytometry instrument for fluorescence-activated cell sorting (FACS) analysis.

### Western blot analysis.

Immortalized hBMECs and iPSC-derived BMECs were infected as described above for 5 h at an MOI of 10. Then, cells were washed three times in sterile PBS, and cell lysates were taken using radioimmunoprecipitation assay (RIPA) buffer plus protease inhibitor cocktail (catalog number 78443; Thermo Scientific). Proteins were quantified using a bicinchoninic acid (BCA) assay (catalog number 23227; Thermo Scientific), and equal amounts were loaded onto protein gels and transferred to nitrocellulose membranes. Anti-COX IV antibody was used as a protein loading control (catalog number 4850T; Cell Signaling Technologies), and anti-claudin-5 antibody (catalog number 35-2500; Thermo Scientific) to visualize claudin-5. Horseradish peroxidase (HRP)-conjugated secondary antibodies (Jackson Laboratory) and a BioRad ChemiDoc XRS+ instrument were used to image the blots.

### Statistics.

GraphPad Prism version 5.0 (GraphPad Software, Inc.) was used for all statistical analysis. For pairwise comparisons, the 2-tailed Student *t* test was used where appropriate. For multiple comparisons, analysis of variance (ANOVA) was used to determine statistics. Data are represented as mean values ± standard errors of the means (SEM) where triplicate mean values are presented and as mean values ± standard deviations (SD) where raw values are presented. Statistical significance was accepted at a *P* value of less than 0.05.
