# Supplementary figures and images for: Modeling Group B Streptococcus and Blood-Brain Barrier Interaction by Using Induced Pluripotent Stem Cell-Derived Brain Endothelial Cells
Source: mSphere. 2017 Nov 1;2(6):e00398-17. doi: 10.1128/mSphere.00398-17 (PMC5663983; doi:10.1128/mSphere.00398-17)

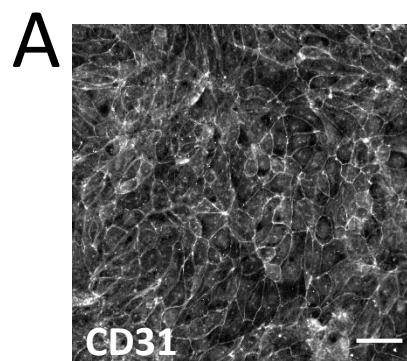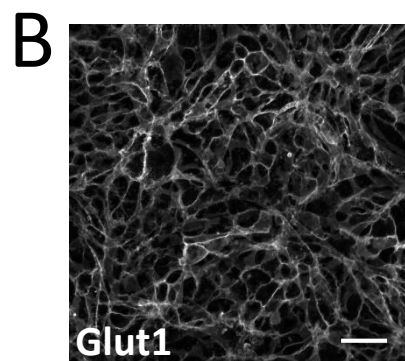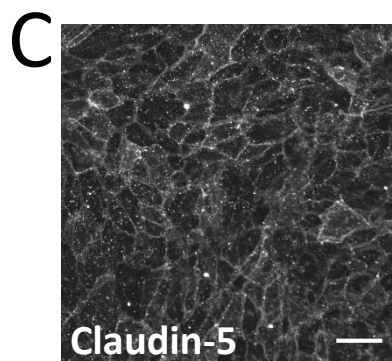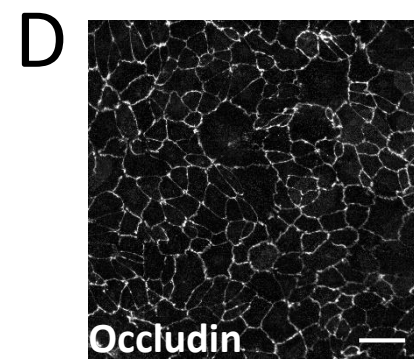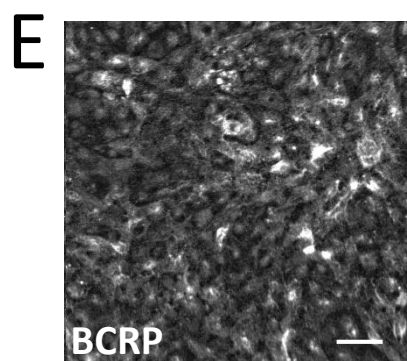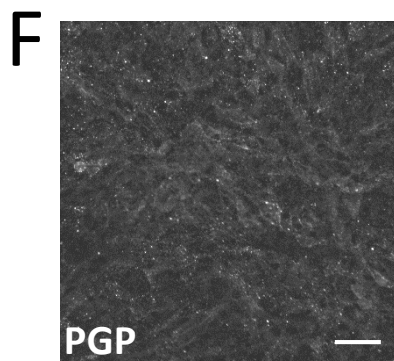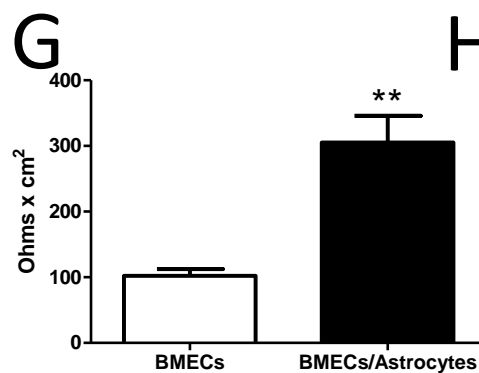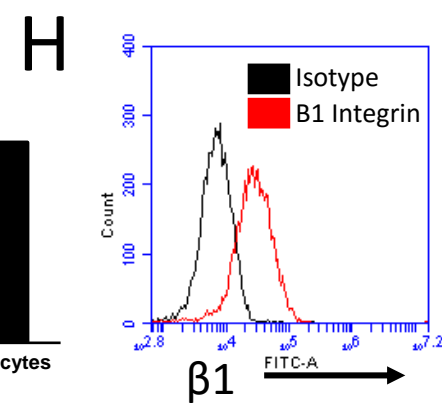

Supplement: FIG S1 [file sph005172391sf1.pdf]

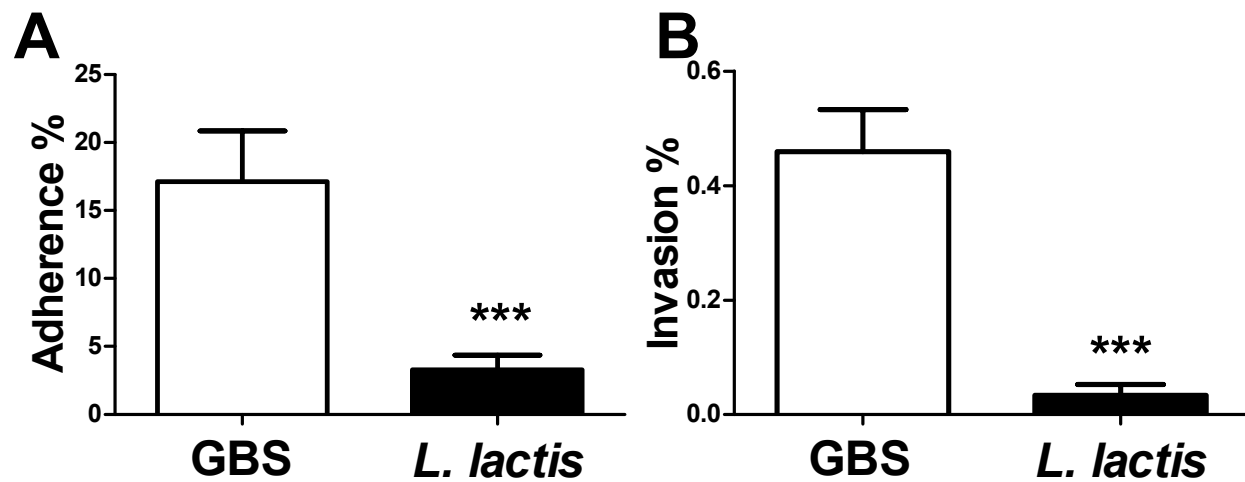

Supplement: FIG S2 [file sph005172391sf2.pdf]

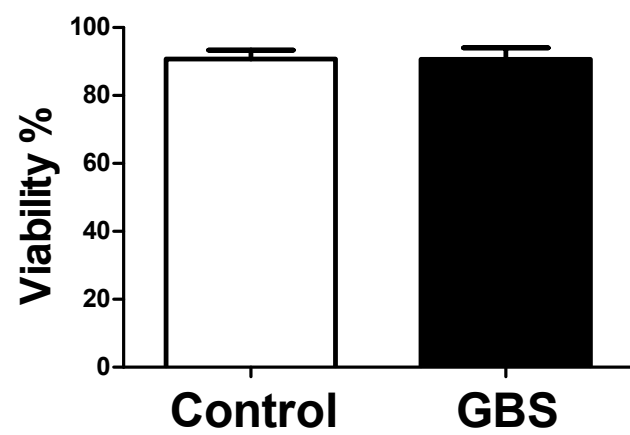

Supplement: FIG S3 [file sph005172391sf3.pdf]

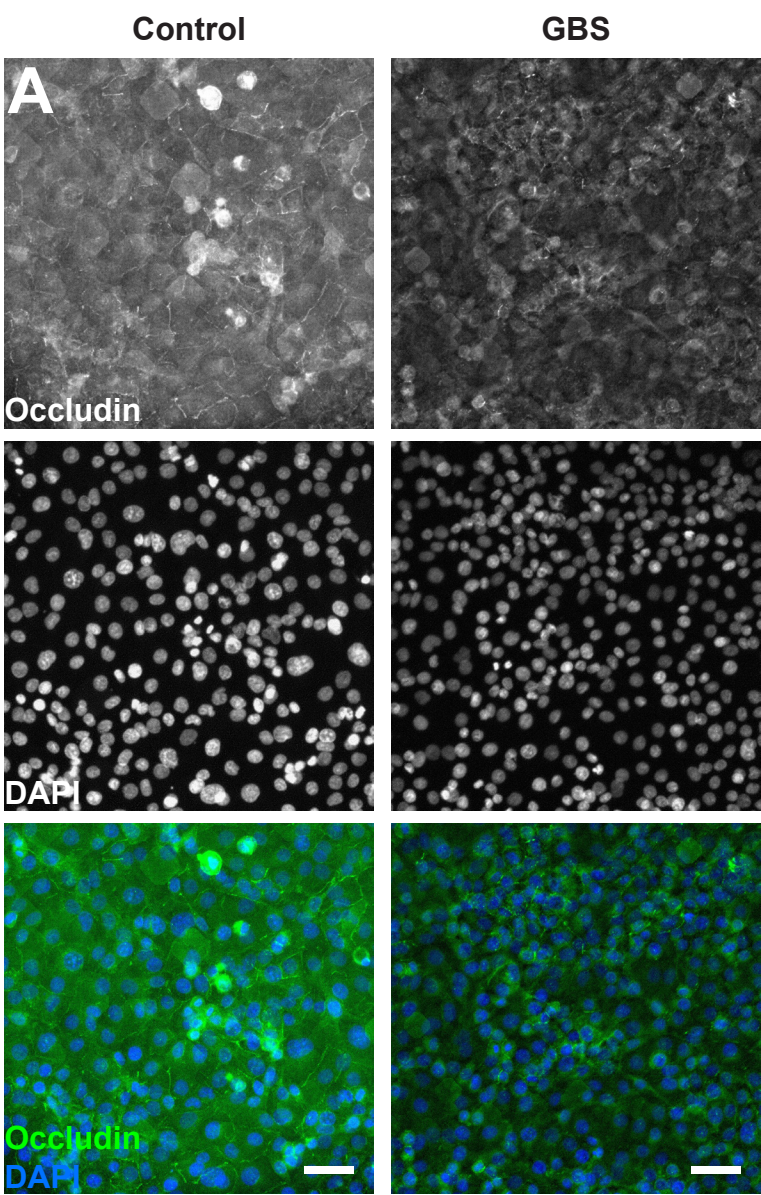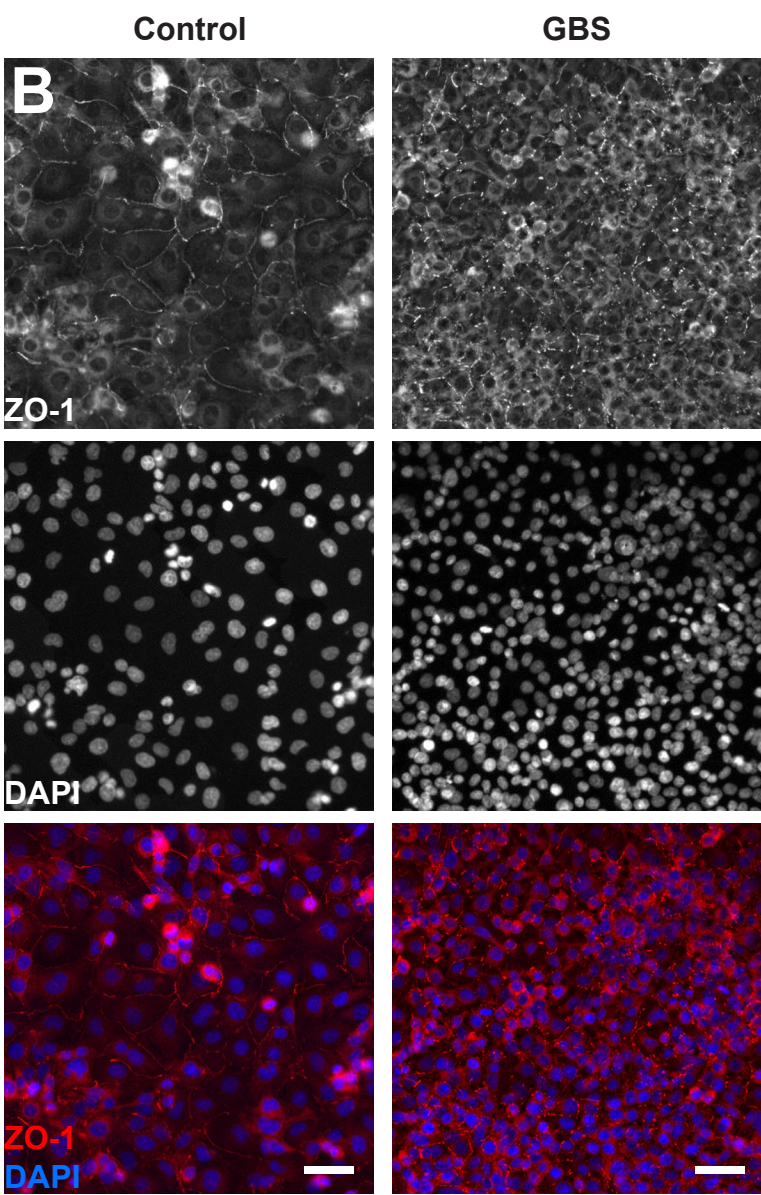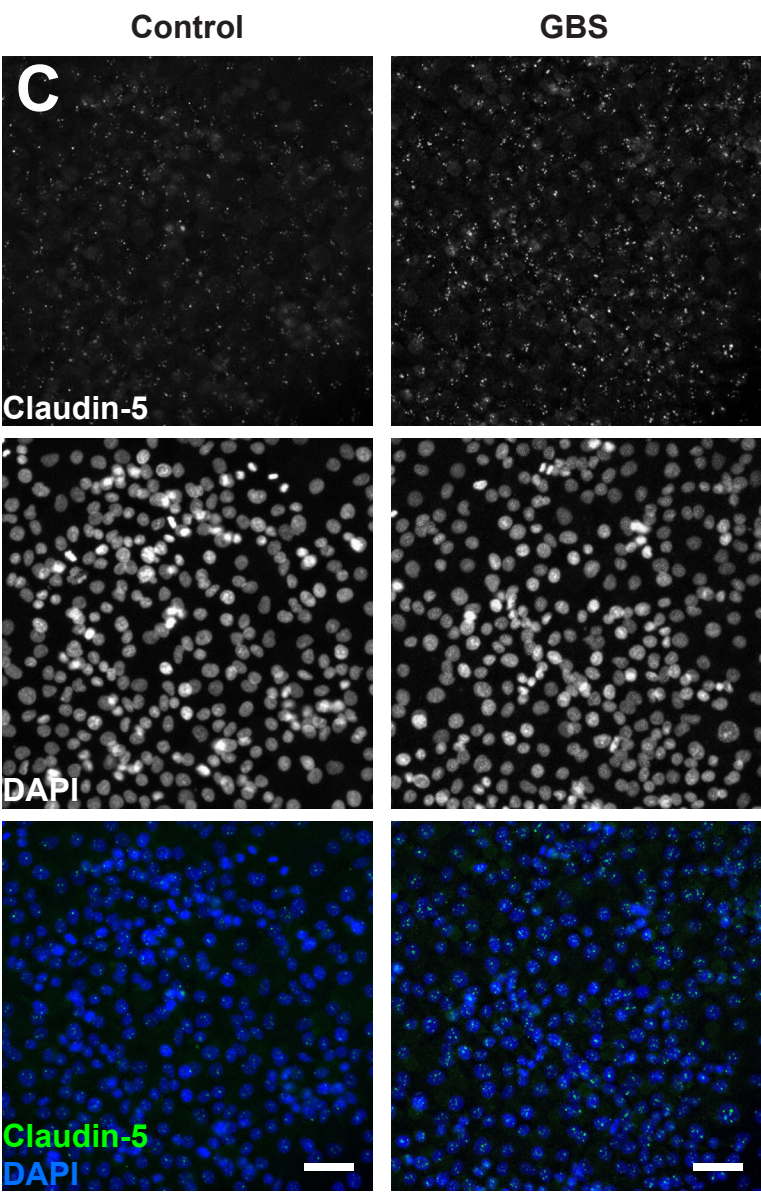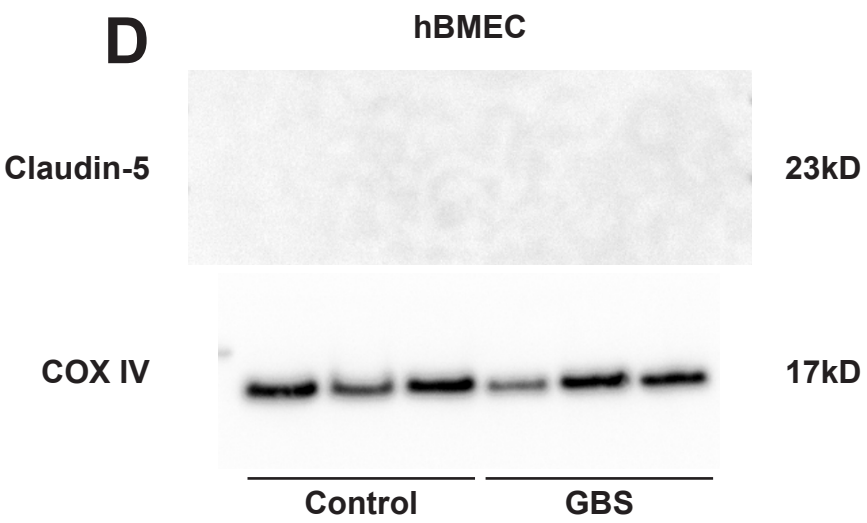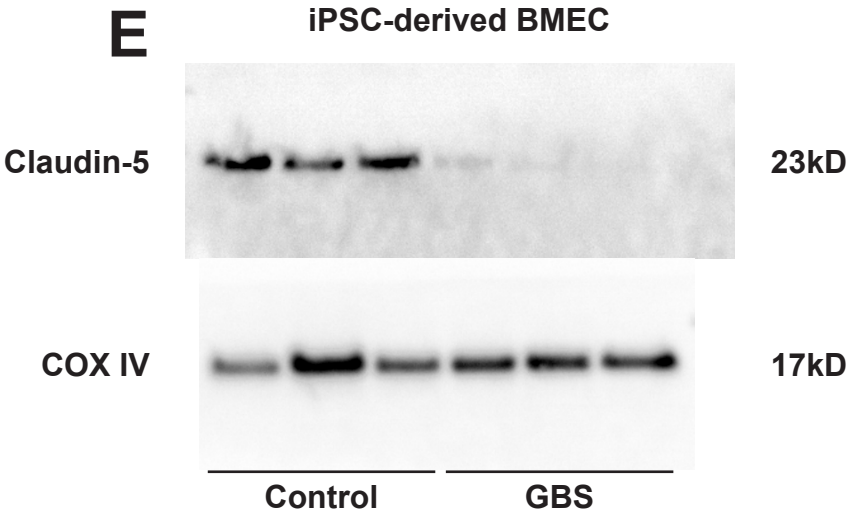

Supplement: FIG S4 [file sph005172391sf4.pdf]
